# Ultrasound-Guided Hydrodissection for Peripheral Neuropathy: An Evidence-Based Intervention Whose Time Has Finally Come

**DOI:** 10.7759/cureus.106578

**Published:** 2026-04-07

**Authors:** King Hei Stanley Lam, Yonghyun Yoon, Daniel Chiung-Jui Su, Teinny Suryadi, Anwar Suhaimi, Yung Tsan Wu

**Affiliations:** 1 Pain Management, Faculty of Medicine, The Chinese University of Hong Kong, New Territories, HKG; 2 Pain Management, Faculty of Medicine, The University of Hong Kong, Hong Kong, HKG; 3 Musculoskeletal Medicine, The Board of Clinical Research, The Hong Kong Institute of Musculoskeletal Medicine, Kowloon, HKG; 4 Orthopaedics, International Academy of Musculoskeletal Medicine, Hong Kong, HKG; 5 Orthopaedics, International Academy of Regenerative Medicine, Incheon, KOR; 6 Orthopaedics, MSKUS, San Diego, USA; 7 Orthopaedic Surgery, Hallym University Kangnam Sacred Heart Hospital, Seoul, KOR; 8 Orthopaedic Surgery, Incheon Terminal Orthopedic Surgery Clinic, Incheon, KOR; 9 Physical Medicine and Rehabilitation, Chi Mei Medical Center, Tainan, TWN; 10 Physical Medicine and Rehabilitation, Medistra Hospital, Jakarta, IDN; 11 Physical Medicine and Rehabilitation, Synergy Clinic, Jakarta, IDN; 12 Physical Medicine and Rehabilitation, Hermina Hospital Podomoro, Jakarta, IDN; 13 Rehabilitation Medicine, Universiti Malaya Medical Centre, Universiti Malaya, Kuala Lumpur, MYS; 14 Physical Medicine and Rehabilitation, Tri-Service General Hospital, Taipei, TWN

**Keywords:** carpal tunnel syndrome, dextrose, hydrodissection, interventional, minimally invasive surgical procedures, nerve compression syndromes, pain management, peripheral nervous system diseases, regenerative medicine, ultrasonography

## Abstract

Peripheral neuropathy remains a prevalent and challenging clinical condition, with management strategies traditionally centered on pharmacologic and neuromodulatory approaches. While these interventions play essential roles, an additional minimally invasive, evidence-based procedure warrants greater recognition: ultrasound-guided perineural hydrodissection.

This editorial presents a contemporary perspective on hydrodissection as a key tool within the peripheral neuropathy treatment algorithm. High-resolution ultrasound enables precise injection of solutions, such as 5% dextrose, to release nerves from surrounding compressive structures. The rationale is supported by animal models demonstrating the vulnerability of nerves to even mild compression, and the therapeutic goal is to restore the function of the nervi nervorum and vasa nervorum through mechanical release and potential metabolic effects. Recent systematic reviews and meta-analyses have established hydrodissection as a safe and effective intervention, particularly for focal entrapment neuropathies such as carpal tunnel syndrome. Its role as a bridge between conservative care and invasive surgery is especially relevant for patients with length-dependent peripheral neuropathies who develop superimposed entrapments. Wider recognition of this technique is essential for providing a complete and contemporary picture of interventional options for refractory neuropathic pain.

## Editorial

Peripheral neuropathy affects approximately 1% of adults worldwide and represents one of the most common neurologic conditions encountered in clinical practice [1]. The management landscape has traditionally been dominated by pharmacologic agents targeting neuropathic pain mechanisms and, for refractory cases, advanced neuromodulatory techniques. While these approaches remain foundational, the evolution of interventional pain medicine has introduced a minimally invasive, evidence-based procedure that deserves greater recognition in contemporary treatment algorithms: ultrasound-guided perineural hydrodissection.

Ultrasound-guided hydrodissection has matured into a safe and effective procedure for treating nerve entrapment [2,3]. The technique involves the injection of a solution, most commonly 5% dextrose in water (D5W) or platelet-rich plasma (PRP)-under high-resolution ultrasound guidance to separate a peripheral nerve from surrounding compressive or adhesive structures, such as fascia, ligaments, or scar tissue [4]. Two principal approaches have been described in the literature, each with distinct technical considerations and learning curves [4].

Method 1

In-Plane Approach With Needle Perpendicular to the Long Axis of the Nerve

In Method 1, the ultrasound probe is positioned perpendicular to the long axis of the nerve (short-axis view), and the needle is advanced in-plane with the transducer, also perpendicular to the long axis of the nerve. The needle first approaches the inferior surface of the nerve with the bevel positioned upward; the pressure of the injectate is used to separate the soft tissues around the nerve, layer by layer, until the injectate surrounds the epineurium. The same process is repeated from the superior aspect of the nerve with the needle bevel positioned downward. This approach allows the injectate to separate the nerve circumferentially from surrounding compressive structures. The hydrodissected nerve appears oval and surrounded by anechoic fluid on ultrasound [4].

**Figure 1:**
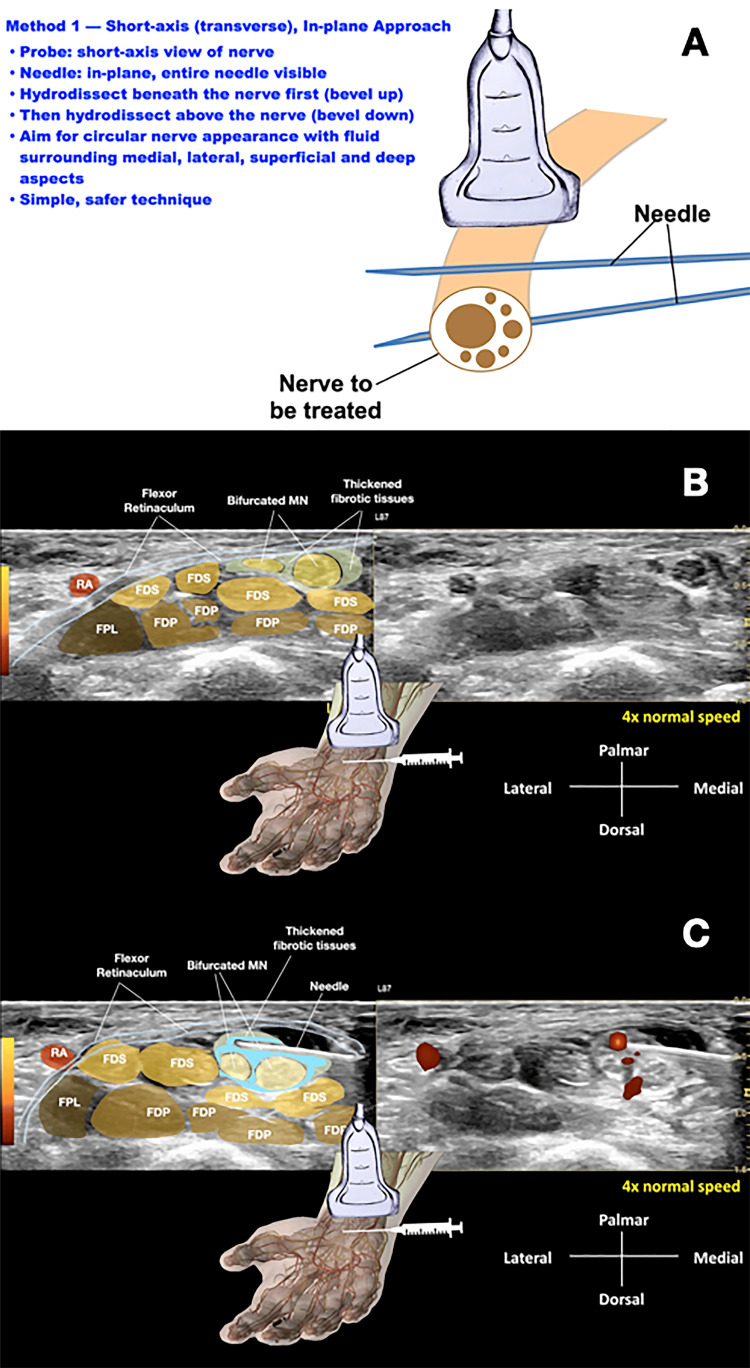
In-plane approach with needle perpendicular to the long axis of the nerve (Method 1) (A) Schematic drawing of Method 1. The ultrasound probe is positioned perpendicular to the long axis of the nerve (short-axis view). The needle is advanced in-plane with the transducer, also perpendicular to the long axis of the nerve, allowing continuous visualization of the entire needle shaft. The needle approaches from the inferior surface with bevel up, then from the superior surface with bevel down, to achieve circumferential release [4]. (B) Ultrasound image of the median nerve (MN) within the carpal tunnel before hydrodissection. The nerve is seen in cross-section with surrounding fascial structures. (C) Ultrasound image after hydrodissection, demonstrating circumferential spread of injectate (as shown by the blue shading) around the median nerve (MN), confirming successful release. Credit: Schematic diagram created in Keynote (Apple Inc., Cupertino, CA, USA). This image was created by Professor Lam using Keynote and was not generated using artificial intelligence. MN, median nerve; FPL, flexor pollicis longus; FDS, flexor digitorum superficialis; FDP, flexor digitorum profundus; RA, radial artery

Figure 1 illustrates this technique. Panel 1A presents a schematic drawing of the general approach, showing the needle and probe both perpendicular to the long axis of the nerve, with the needle visualized in-plane. Panel 1B shows the median nerve within the carpal tunnel before hydrodissection. Panel 1C demonstrates the injectate surrounding the median nerve following hydrodissection, achieving the desired circumferential release.

**Video 1:** Ultrasound-guided hydrodissection of the median nerve using Method 1 - short-axis (transverse), in-plane Real-time demonstration of hydrodissection with the probe in short-axis (perpendicular to the nerve). The needle is advanced in-plane, allowing continuous visualization of the shaft and tip. First, the needle approaches the inferior surface of the nerve with the bevel up, and injectate is delivered to separate tissue layers. The step is then repeated from the superior surface with the bevel down. Successful hydrodissection is confirmed by the circumferential spread of injectate around the nerve, which achieves a circular appearance. Credit: Video courtesy of Professor King Hei Stanley Lam. MN, median nerve; FPL, flexor pollicis longus; FDS, flexor digitorum superficialis; FDP, flexor digitorum profundus; RA, radial artery

Method 2

Out-of-Plane With Subsequent In-Plane Approach

In Method 2, the needle is positioned parallel to the long axis of the nerve, and the approach is performed in two stages. First, with the probe perpendicular to the long axis of the nerve (short-axis view), an out-of-plane technique is used to hydrodissect the nerve from surrounding tissues. The injectate is delivered to surround the nerve circumferentially, confirming that the nerve is freed from surrounding soft tissues by visualization of anechoic fluid both above and below the nerve. Subsequently, the probe is turned parallel to the long axis of the nerve (long-axis view), and the needle tip is guided back to the proximal aspect of the nerve using an in-plane technique. Fluid is injected above the nerve, with the bevel positioned downward to avoid accidental contact, and the injectate is visualized tracking along the longitudinal axis of the nerve [4].

**Figure 2:**
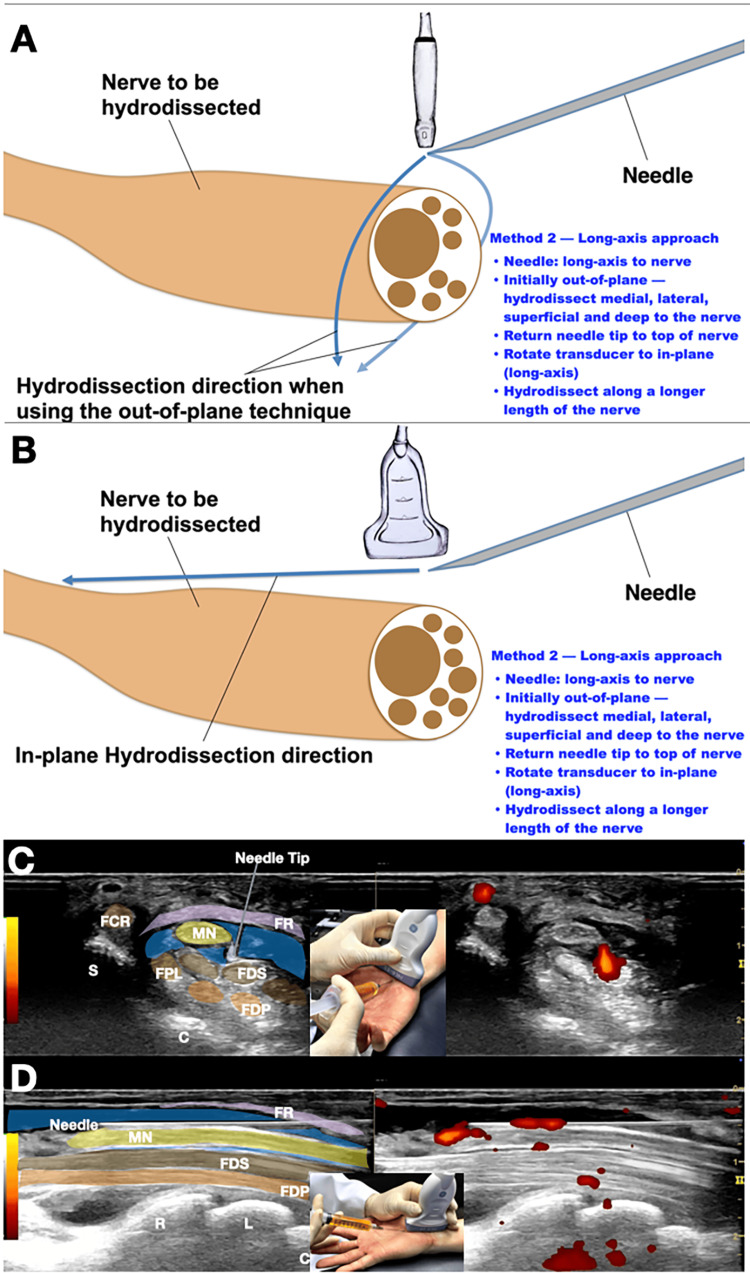
Initial out‑of‑plane hydrodissection followed by in‑plane (long‑axis) needle placement (Method 2) (A) Schematic drawing of Step 1: the probe is positioned perpendicular to the long axis of the nerve (short-axis view). The needle is parallel to the nerve (out-of-plane technique) to achieve initial circumferential release [4]. (B) Schematic drawing of Step 2: the probe is turned parallel to the long axis of the nerve (long-axis view). The needle remains parallel to the nerve, now visualized in-plane, allowing hydrodissection to continue along the longitudinal axis [4]. (C) Ultrasound image captured from Video 2, showing out-of-plane hydrodissection of the MN with circumferential injectate spread (as shown by the blue shading). (D) Ultrasound image captured from Video 2, showing in-plane hydrodissection of the MN, with longitudinal injectate spread (as shown by the blue shading) along the nerve's axis. Credit: Schematic diagram created in Keynote (Apple Inc., Cupertino, CA, USA). This image was created by Professor Lam using Keynote and was not generated using artificial intelligence. MN, median nerve; FPL, flexor pollicis longus; FDS, flexor digitorum superficialis; FDP, flexor digitorum profundus; FR, flexor retinaculum; C, capitate; S, scaphoid; R, radius; L, lunate

Figure 2 illustrates this approach. Panel 2A is a schematic drawing showing the first step: the probe is perpendicular to the nerve (short-axis view), and the needle is parallel to the nerve, using an out-of-plane technique to achieve initial circumferential release. Panel 2B depicts the second step: the probe is turned parallel to the nerve (long-axis view), and the needle remains parallel, now visualized in-plane, allowing continued hydrodissection along the longitudinal axis. Panel 2C presents an ultrasound image captured from Video 2, demonstrating the out-of-plane hydrodissection of the median nerve, with circumferential injectate spread. Panel 2D shows the in-plane hydrodissection of the median nerve, illustrating the longitudinal spread of injectate along the nerve (Video 2).

**Video 2:** Median nerve hydrodissection using Method 2 - out-of-plane hydrodissection followed by in-plane long-axis placement This video demonstrates the stepwise approach to Method 2. The procedure begins with the probe positioned perpendicular to the long axis of the nerve (short-axis view). The needle is advanced parallel to the nerve, using an out-of-plane technique to achieve initial circumferential release, confirmed by visualization of anechoic fluid surrounding the nerve. The needle tip is then repositioned to the superior aspect of the nerve; the probe is rotated to align parallel to the long axis of the nerve (long-axis view), and the needle tip is guided to hydrodissect back to the proximal aspect of the nerve using an in-plane technique. Fluid is injected above the nerve, with the bevel positioned downward, and the injectate is visualized tracking longitudinally along the nerve, both above and below. This combined approach achieves both circumferential and longitudinal release over a longer segment. Credit: Video courtesy of Professor King Hei Stanley Lam. MN, median nerve; FDS, flexor digitorum superficialis; FDP, flexor digitorum profundus

Regardless of the approach selected, the fundamental principle remains that the fluid injectate - not the needle tip - performs the critical work of dissecting and separating the soft tissues [4]. Slow needle advancement while injecting minimizes disruption of important soft tissues, such as vascular bundles and adjacent nerves, and maximizes patient comfort by allowing surrounding tissues to be gently pushed aside by a continuous fluid jet [4].

The rationale for hydrodissection is deeply rooted in fundamental pathophysiology. Bennett's animal model of neuropathic pain demonstrated that even mild, non-ischemic nerve constriction can induce rapid morphological changes and persistent allodynia, highlighting the marked vulnerability of peripheral nerves to light compression [5]. Subsequent studies have confirmed that perineural adhesions and increased intraneural pressure can compromise both axonal transport and microcirculation, forming the pathophysiological basis for targeted mechanical release [6,7]. The therapeutic objective of hydrodissection is to reverse this process, releasing the nerve to restore function to the nervi nervorum and improve microcirculation by decompressing the vasa nervorum [4,8].

The clinical evidence supporting hydrodissection has expanded considerably in recent years, positioning it as a standard component of the treatment algorithm for focal entrapment neuropathies. A 2025 review in Muscle & Nerve explicitly positioned hydrodissection as a key intervention following failed conservative management (e.g., splinting, therapy) but prior to the consideration of surgical release, thereby filling a critical therapeutic gap [3]. Randomized controlled trials have demonstrated superior improvements in symptom severity and functional status with D5W hydrodissection compared to traditional corticosteroid injections at intermediate follow-up periods [9,10]. A 2023 systematic review and meta-analysis in diagnostics synthesized evidence from multiple randomized controlled trials and concluded that ultrasound-guided hydrodissection with D5W provides clinically important and durable benefits for carpal tunnel syndrome, with efficacy at six months comparable to corticosteroid injection and superior to placebo [2]. A 2024 network meta-analysis further confirmed these findings, demonstrating that D5W hydrodissection ranked favorably among available injectable treatments for carpal tunnel syndrome [11], and a 2025 meta-analysis substantiated the comparable efficacy of D5W to corticosteroid injections with a more favorable safety profile [12]. The proposed mechanisms of action extend beyond the mechanical benefits of release, with hypotheses suggesting a direct modulatory effect of dextrose through the downregulation of the TRPV1 ion channel (implicated in chronic neuropathic pain) and the correction of perineural glycopenia, which can lead to nerve hyperexcitability [4,13].

While the strongest evidence currently exists for focal entrapment syndromes, the therapeutic principle is directly applicable to the broader patient population with peripheral neuropathy. It is a common clinical scenario for patients with length-dependent peripheral neuropathies, such as those secondary to diabetes, to develop superimposed, focal nerve compressions at sites of anatomic vulnerability (e.g., the common peroneal nerve at the fibular head or the tibial nerve within the tarsal tunnel) [3,13-15]. For these patients, ultrasound-guided hydrodissection represents a logical and minimally invasive intervention. It serves as a critical bridge between systemic pharmacologic management and more invasive surgical or neuromodulation techniques, offering a targeted approach to a superimposed mechanical problem [3,16].

In summary, ultrasound-guided perineural hydrodissection represents a safe, effective, and increasingly evidence-based intervention for peripheral neuropathies, particularly those with a compressive component. As the quality and volume of evidence continue to accumulate, this technique should no longer be regarded as experimental. Its integration into clinical practice and inclusion in future discourse on peripheral neuropathy management are essential to provide clinicians with a complete and contemporary picture of interventional options for improving patient outcomes and addressing refractory neuropathic pain.
